# Multi-Omics and Single-Cell Mendelian Randomization Reveal a Potential Role of *VNN2* in Lung Adenocarcinoma in Resting Natural Killer Cells

**DOI:** 10.14740/wjon2689

**Published:** 2026-03-05

**Authors:** Zhi Chun Xue, Jing Lin, Jian Hui Wu, Zhi Wen Peng, Mei Yan Tang, Peng Liang, Hui Ling Chen, Gui Ju Fang, Qing Xue

**Affiliations:** a Department of Respiratory and Critical Care Medicine, Ningde Municipal Hospital of Ningde Normal University, Ningde, Fujian, China; b Department of Respiratory and Critical Care Medicine, Ningde Clinical Medical College of Fujian Medical University, Ningde, Fujian, China; c Department of Emergency Medicine, Ningde Municipal Hospital of Ningde Normal University, Ningde, Fujian, China; d College of Marine Sciences, Ningde Normal University, Ningde, Fujian, China

**Keywords:** *VNN2*, Lung adenocarcinoma, Mendelian randomization, sc-eQTL, Resting NK cells

## Abstract

**Background:**

We aimed to evaluate the potential association between genetically predicted vanin-2 (*VNN2*) expression and lung adenocarcinoma (LUAD) risk, and to explore the immune cell subtype that may underlie this relationship.

**Methods:**

We integrated whole-blood expression quantitative trait loci (eQTL) data from eQTLGen, plasma protein quantitative trait loci (pQTL) data from deCODE, and LUAD genome-wide association study (GWAS) data from European-ancestry cohorts, together with differential expression analysis using GEPIA2, to identify candidate genes for subsequent single-cell eQTL (sc-eQTL) Mendelian randomization (MR) analysis. For the sc-eQTL analysis, *VNN2*-associated eQTLs from 14 immune cell types profiled in the OneK1K single-cell eQTL resource were tested for associations with LUAD risk.

**Results:**

Bulk-level MR analysis showed that genetically predicted increases in *VNN2* expression and protein levels were significantly associated with a reduced risk of LUAD (eQTL-MR: odds ratio (OR) = 0.964, 95% confidence interval (95% CI), 0.934–0.995; P = 0.024; pQTL-MR: OR = 0.946, 95% CI, 0.921–0.970; P = 2.87 × 10^−5^). Transcriptomic analyses confirmed significant downregulation of VNN2 in LUAD tumors compared with normal lung tissues. sc-eQTL MR identified the strongest association in resting natural killer (rNK) cells (OR = 0.896, 95% CI, 0.829–0.967; P = 0.005).

**Conclusions:**

Multi-omics and sc-eQTL MR analyses indicated that genetically predicted increases in *VNN2* expression were associated with a reduced risk of LUAD, with the most pronounced effect observed in rNK cells. These findings suggest a potential cell type–specific role of *VNN2* in LUAD susceptibility and warrant further studies to validate its biological relevance and clinical implications.

## Introduction

Lung adenocarcinoma (LUAD), the most common histologic subtype of lung cancer, remains a leading cause of cancer-related mortality worldwide [1, 2]. Despite diagnostic and therapeutic advances, the global incidence of LUAD continues to increase, and the 5-year survival rate remains low, especially among patients diagnosed at an advanced stage [3]. These observations underscore the urgent need to elucidate the molecular mechanisms underlying LUAD and to identify novel biomarkers for early detection and personalized therapy [4].

The tumor microenvironment (TME) plays a pivotal role in cancer progression, with immune cells, particularly natural killer (NK) cells, acting as key mediators of tumor surveillance and immune defense [5]. Among these subsets, resting natural killer (rNK) cells have gained increasing attention due to their roles in maintaining immune homeostasis and mediating early antitumor responses. However, the molecular mechanisms that regulate their function in LUAD remain poorly understood [6].

*Vanin-2* (*VNN2*) encodes a glycosylphosphatidylinositol (GPI)-anchored membrane protein involved in inflammation, immune regulation, and hematopoietic functions [7, 8]. Although *VNN2* has been proposed as a biomarker for immune-related and metabolic disorders, its role within the TME remains largely unexplored [9]. Most transcriptomic and expression quantitative trait locus (eQTL) studies of the TME have relied on bulk-level analyses, which lack the resolution to distinguish cell type–specific regulatory effects and may therefore overlook the contributions of immune cell heterogeneity to tumor progression [10, 11]. Recent advances in single-cell RNA sequencing (scRNA-seq) and single-cell eQTL (sc-eQTL) mapping offer powerful means to dissect cell type–specific regulatory mechanisms [12]. However, no direct evidence has linked the regulation of *VNN2* expression within rNK cells to LUAD risk.

Recent sc-eQTL studies utilizing large-scale single-cell transcriptomic datasets have uncovered cell type–specific regulatory effects that are often overlooked in bulk analyses [13, 14]. In parallel, MR which integrates multi-omics data sources such as eQTL, pQTL, and genome-wide association study (GWAS) data, provides a powerful framework for inferring potential causal links between gene expression and disease risk [15]. Integrating sc-eQTL data with MR and colocalization analyses enables systematic investigation of cell type–specific genetic mechanisms at single-cell resolution [16, 17]. However, this integrative approach has not yet been applied to evaluate whether *VNN2* expression within rNK cells is associated with the risk of LUAD.

In this study, we integrated sc-eQTL and Mendelian randomization (MR) analyses to evaluate whether genetically predicted *VNN2* expression is associated with LUAD risk among different immune cell subtypes. This approach enabled us to explore the cell type–specific roles of *VNN2* in immune regulation within the TME and to evaluate its potential relevance as a biomarker or therapeutic target. Given the emerging evidence of rNK cell involvement in LUAD, we speculated that any association would be most evident in rNK cells.

## Materials and Methods

### Study design

**Figure 1:**
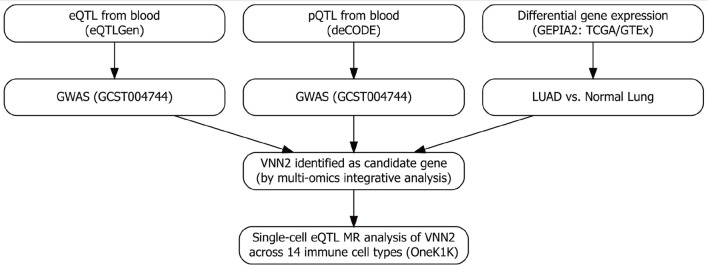
Overview of the multi-omics Mendelian randomization (MR) framework. Stage 1: genome-wide screening integrating whole-blood eQTL (eQTLGen), plasma pQTL (deCODE), LUAD GWAS, and transcriptomic differential expression analyses (TCGA and GTEx via GEPIA2) to prioritize candidate genes. Stage 2: targeted single-cell MR analyses using the OneK1K sc-eQTL resource to evaluate cell type–specific associations with lung adenocarcinoma (LUAD) risk, with a focus on resting NK cells. eQTL: expression quantitative trait loci; NK: natural killer.

To clarify the analytical logic, we adopted a two-stage integrative MR framework to investigate the genetic determinants of LUAD using multi-omics data (Fig. 1). Stage 1 (bulk-level discovery): Whole-blood eQTL data from the eQTLGen Consortium and plasma protein pQTL data from the deCODE Genetics project were used as exposure datasets, while LUAD GWAS summary statistics (accession GCST004744) served as the outcome dataset. Multi-omics MR analyses were performed to identify genes whose genetically proxied expression or protein levels were associated with LUAD risk. To verify the transcriptomic direction and biological plausibility of these associations, GEPIA2—which integrates TCGA and GTEx transcriptomic data—was used to compare gene expression between LUAD tumor and normal tissues and to assess their prognostic relevance. Stage 2 (single-cell resolution): Genes identified from stage 1 were further evaluated using single-cell eQTL (sc-eQTL) data from the OneK1K project, which profiles genetic effects on gene expression across 14 peripheral immune cell types. Cell type–specific MR analyses were conducted to localize the bulk-level associations to specific immune subsets and to clarify potential immune-cell–mediated regulatory mechanisms. MR analyses were conducted under three core assumptions [18]: genetic instruments were strongly associated with the exposure; instruments were independent of both known and unknown confounders; and instruments influenced the outcome exclusively through the exposure, without horizontal pleiotropy.

### Data sources and ethical statement

Five complementary public resources were integrated to support the two-stage MR framework: eQTLGen [19] provided genome-wide cis-eQTL summary data for whole blood and served as the transcriptomic exposure layer [20]. deCODE Genetics [21] offered plasma pQTL summary data, representing the proteomic exposure layer [22]. GWAS Catalog (LUAD accession GCST004744 [23]) provided the disease outcome data (11,273 LUAD cases and 55,483 controls; total n = 66,756) [24]. GEPIA2 [25] integrated TCGA and GTEx expression data to validate differential expression and survival associations of candidate genes. OneK1K project [26] offered single-cell eQTL data from 1.27 million peripheral blood mononuclear cells across 14 immune cell types, enabling cell type–specific MR analyses. Collectively, eQTLGen and deCODE were used to detect causal signals at the bulk level, GWAS data provided the LUAD outcome, GEPIA2 confirmed expression directionality and clinical relevance, and OneK1K refined these associations to specific immune subsets. All datasets were publicly available and de-identified, with ethics approval granted by the respective institutional review boards of the original studies. A detailed summary of dataset characteristics and analytical roles is provided in Supplementary Material 1 (wjon.elmerpub.com).

### Instrument selection and quality control

Single nucleotide polymorphisms (SNPs) that met the genome-wide significance threshold (P < 5 × 10^−8^) were selected as instrumental variables (IVs) using a clumping window of 10,000 kb and a linkage disequilibrium (LD) threshold of r^2^ < 0.1. To minimize weak instrument bias, only SNPs with an F-statistic > 10 were retained. Palindromic and ambiguous SNPs were excluded from the analysis. All IVs were rigorously screened, which included removing SNPs associated with known confounders or outcome traits identified using the LDtrait tool [27] and the GWAS Catalog [28].

### Differential expression analysis

Transcriptomic data from LUAD tumor and matched normal lung tissues were analyzed via the GEPIA2 platform. Differentially expressed genes were identified based on an absolute log_2_ fold change (|log_2_FC|) > 1 and a false discovery rate (FDR)-adjusted P < 0.05 [29].

### Candidate gene prioritization

Candidate genes were prioritized through the integration of multi-omics evidence, including differential expression profiles, eQTL/pQTL associations, and MR estimates, emphasizing genes consistently supported across multiple analytical layers. Genes whose genetically predicted higher expression was associated with increased LUAD risk and that were upregulated in tumors were classified as putative oncogenes, whereas genes showing the opposite direction of association were considered putative tumor suppressors. This approach highlights candidates showing concordant directions between MR estimates and transcriptomic expression patterns, thereby prioritizing biologically meaningful targets.

### Extraction and comparative analysis of sc-eQTLs

Following the identification of candidate genes through multi-omics genome-wide screening, we systematically characterized their genetic regulatory architecture across immune cell subpopulations. For each candidate gene, eQTL signals were retrieved from sc-eQTL summary statistics spanning 14 distinct immune cell types. The summary statistics for each cell type were iteratively processed to extract all SNP–gene pairs associated with the candidate genes. SNPs with nominal eQTL P < 0.05 were retained to capture potential regulatory variants. To ensure variant independence, linkage disequilibrium (LD) pruning (r^2^ < 0.1) was applied to remove highly correlated SNPs, resulting in the final set of independent eQTLs for each immune cell type.

### MR analysis

Two-sample MR analyses were performed using the TwoSampleMR R package [18]. After rigorous quality control, bulk eQTL, pQTL, and sc-eQTL datasets were used as exposure datasets, with LUAD GWAS summary statistics as the outcome dataset. Causal estimates were obtained using the Wald ratio method for single-SNP instruments, whereas the inverse-variance weighted (IVW) approach was applied as the primary method for exposures with multiple SNP instruments. To account for potential horizontal pleiotropy, several robust MR methods were additionally implemented, including MR-Egger regression, the weighted median, simple mode, and weighted mode, to evaluate the robustness of causal estimates under different model assumptions. Horizontal pleiotropy was formally evaluated using the MR-Egger intercept test, whereas heterogeneity was assessed using Cochran’s Q statistic. Leave-one-out (LOO) analyses were further performed within the IVW framework. In each iteration, one SNP was removed from the instrument set to examine whether the overall causal estimate was disproportionately influenced by any single variant.

### Transcriptomic and single-cell validation

Differential expression and survival analyses of *VNN2* were conducted via the GEPIA2 platform [25], integrating TCGA and GTEx datasets. The Expression and Stage Plot modules were used to analyze differential expression and stage-specific variation of *VNN2* across LUAD pathological stages (I–IV). The Survival Analysis module was used to assess the prognostic relevance of *VNN2* expression based on Kaplan–Meier survival curves. To further investigate the cellular distribution of *VNN2* within the TME, scRNA-seq data from the NSCLC dataset (GSE148071) were obtained from the TISCH2 database [30]. Violin plots generated within TISCH2 were used to depict cell type–specific expression levels across major immune and stromal cell populations.

### Ethical statements

The authors are accountable for all aspects of the work in ensuring that questions related to the accuracy or integrity of any part of the work are appropriately investigated and resolved. This study was conducted using publicly available summary-level data from genome-wide association studies (GWAS) and expression quantitative trait loci (eQTL), protein quantitative trait loci (pQTL), and single-cell eQTL (sc-eQTL) databases. All data sources used in this analysis obtained ethical approval from their respective institutional review boards, and no individual-level data were used.

## Results

### Genome-wide MR analysis identifies candidate genes associated with LUAD risk

**Figure 2:**
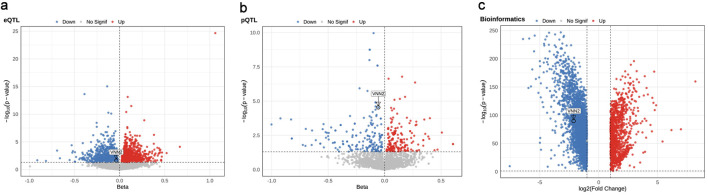
Multi-omics identification of candidate genes associated with lung adenocarcinoma (LUAD). (a) eQTL-MR, (b) pQTL-MR, and (c) transcriptomic differential expression volcano plots highlight genes associated with LUAD risk.

To systematically evaluate the genetic determinants of LUAD, we integrated whole-blood eQTL, plasma pQTL, and European-ancestry LUAD GWAS summary statistics to perform genome-wide MR analyses. In the eQTL-based MR analysis, 1,798 genes showed nominally significant associations with LUAD risk (P < 0.05). Among these, 911 (50.7%) had positive β coefficients, indicating increased risk, whereas 887 (49.3%) had negative β coefficients, indicating reduced risk (Fig. 2a and Supplementary Material 2, wjon.elmerpub.com). In the pQTL-based MR analysis, 241 proteins were associated with LUAD risk at P < 0.05, including 127 (52.7%) with positive β coefficients and 114 (47.3%) with negative β coefficients (Fig. 2b and Supplementary Material 3, wjon.elmerpub.com).

### Differential expression in LUAD tissues

Differential gene expression analysis was performed using the GEPIA2 platform, which integrates transcriptomic data from The Cancer Genome Atlas (TCGA) and the Genotype-Tissue Expression (GTEx) project. A total of 4,245 genes were differentially expressed (|log_2_FC| > 1, FDR < 0.05), including 1,109 upregulated and 3,136 downregulated genes (Fig. 2c and Supplementary Material 3, wjon.elmerpub.com). Transcriptomic analysis revealed markedly reduced *VNN2* mRNA expression in LUAD tumors relative to adjacent normal lung tissues (log_2_FC = –2.13, P = 1.55 × 10^−90^; Supplementary Material 4, wjon.elmerpub.com).

### Cross-omics integration of candidate genes

To identify genes supported by convergent multi-omics evidence, we integrated results from eQTL-based MR, pQTL-based MR, and transcriptomic differential expression analyses. *VNN2* showed consistent evidence across the three analyses. Transcriptomic analysis confirmed markedly reduced *VNN2* mRNA expression in LUAD tumors relative to adjacent normal lung tissues (log_2_FC = –2.13, P = 1.55 × 10^−90^; Supplementary Material 5, wjon.elmerpub.com). In eQTL-based MR analysis, genetically predicted higher *VNN2* expression was significantly associated with reduced LUAD risk (β = –0.036, OR = 0.964, 95% CI: 0.934–0.995, P = 0.024; Supplementary Material 2, wjon.elmerpub.com). Consistent findings were obtained in pQTL-based MR analysis, in which genetically predicted higher *VNN2* protein levels were significantly associated with reduced LUAD risk (β = –0.056, OR = 0.946, 95% CI: 0.921–0.970, P = 2.87 × 10^−5^; Supplementary Material 3, wjon.elmerpub.com).

### Cell type–specific MR analysis of *VNN2* in peripheral immune cell subsets

**Figure 3:**
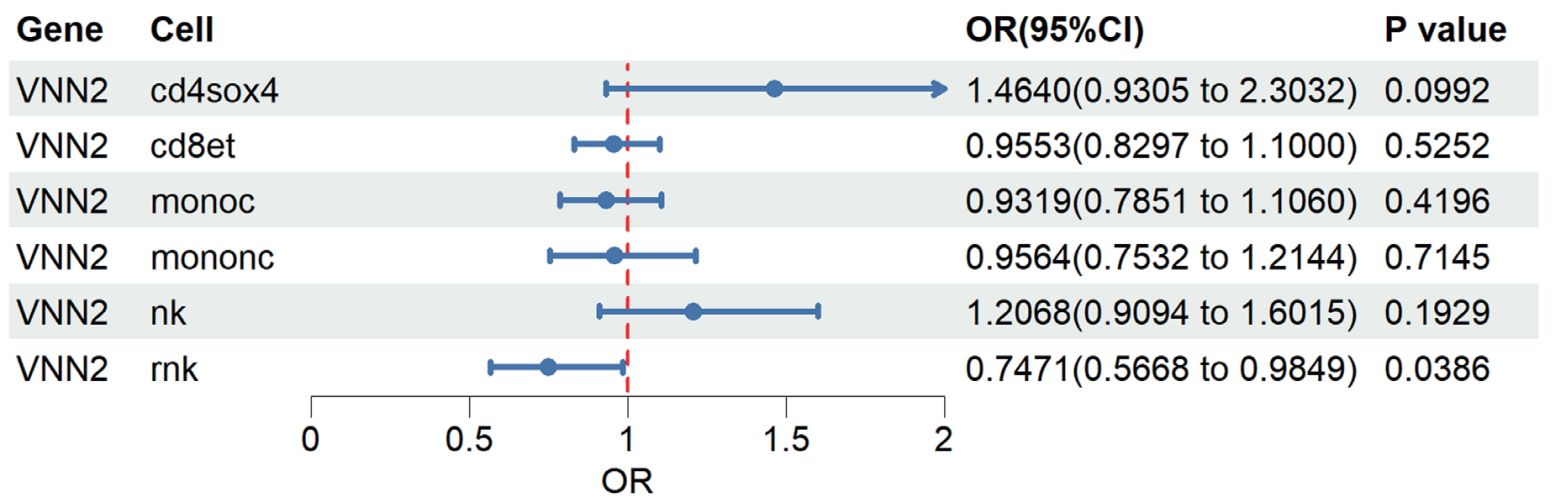
Single-cell Mendelian randomization (MR) analysis of *VNN2* across immune cell types. Overview of the OneK1K dataset comprising 14 peripheral blood immune cell types profiled for eQTLs. Forest plot showing MR estimates of genetically predicted *VNN2* expression and lung adenocarcinoma (LUAD) risk across immune cell subsets. eQTL: expression quantitative trait loci.

To investigate whether the genetic association between *VNN2* expression and LUAD risk exhibits cell type–specific effects, we performed single-cell eQTL–based MR analysis across 14 peripheral blood immune cell types. In the OneK1K dataset, cis-eQTLs for *VNN2* meeting genome-wide significance and instrument strength thresholds were identified in only six immune cell types—CD4^+^SOX4^+^ T, CD8^+^ effector T, classical monocytes, nonclassical monocytes, NK cells, and rNK cells. In the remaining eight immune cell types, no significant *VNN2*-associated SNPs were detected, indicating the absence of valid genetic instruments for MR analysis. For each cell type, only independent eQTLs (P < 0.05 after LD clumping) were retained as instrumental variables. Genetically predicted higher *VNN2* expression in rNK cells was significantly associated with reduced LUAD risk (OR = 0.75, 95% CI: 0.57–0.98, P = 0.039). No statistically significant associations were observed in other immune cell types (Fig. 3 and Supplementary Material 5, wjon.elmerpub.com).

### Sensitivity analyses

To evaluate the robustness of the MR findings, we performed a series of sensitivity analyses. Using robust estimators such as MR-Egger regression, weighted median, and weighted mode, the causal estimates for *VNN2* expression and LUAD risk were directionally consistent with those obtained from the IVW analysis, supporting the robustness of the causal inference (Supplementary Material 6, wjon.elmerpub.com). The MR-Egger intercept test showed no evidence of directional pleiotropy, and Cochran’s Q statistic indicated no substantial heterogeneity among the SNP instruments. Moreover, LOO analyses showed that excluding any single SNP did not disproportionately alter the IVW estimates for either eQTL- or pQTL-based analyses (Supplementary Material 7, wjon.elmerpub.com), confirming that no single variant drove the observed associations.

We further evaluated the expression pattern and clinical relevance of *VNN2* using publicly available transcriptomic and single-cell datasets. Kaplan–Meier survival analysis using the GEPIA2 database showed no significant difference in overall survival between patients with high and low *VNN2* expression (log-rank P = 0.66, hazard ratio (HR) = 0.94; Supplementary Material 8, wjon.elmerpub.com). Stage-specific analysis using the GEPIA2 Stage Plot module indicated no significant differences in *VNN2* expression among pathological stages I–IV of LUAD (analysis of variance (ANOVA) P = 0.964; Supplementary Material 9, wjon.elmerpub.com). To visualize the cellular distribution of *VNN2* within the TME, we examined the non-small cell lung cancer (NSCLC) scRNA-seq dataset (GSE148071) available in the TISCH2 database. As shown in Supplementary Material 10 (wjon.elmerpub.com), *VNN2* expression was relatively higher in immune cell populations—particularly monocytes and macrophages—while being nearly absent in epithelial and malignant cells. Notably, although rNK cells were not individually annotated in this dataset, the overall immune cell–restricted expression pattern of *VNN2* was consistent with its proposed immunoregulatory role in the TME.

## Discussion

In this study, we systematically integrated multi-omics MR analyses with sc-eQTL data to investigate genetic associations linking *VNN2* expression with LUAD risk. Our analyses showed that higher genetically predicted *VNN2* expression was significantly associated with reduced LUAD risk at both transcriptomic and proteomic levels, consistent with its downregulation in LUAD tumor tissues relative to normal lung tissues. The detection of *VNN2* in plasma likely reflects its soluble, cleaved form released from leukocyte membranes, as previously reported for phospholipase-mediated GPI-anchor hydrolysis. Furthermore, single-cell MR analysis showed that this association was most pronounced in rNK cells, suggesting a potential cell type–specific involvement in tumor immunity. These findings extend previous bulk-level omics observations to single-cell resolution and provide exploratory genetic evidence that may inform future mechanistic studies and NK cell–targeted therapeutic strategies.

Bulk transcriptomic analysis (TCGA/GTEx via GEPIA2) indicated lower *VNN2* expression in LUAD tumor tissues relative to normal lung tissues; however, this pattern should not be interpreted as indicating that tumor cells are the principal source of *VNN2*. *VNN2* (Vanin-2, GPI-80) is a GPI-anchored membrane protein primarily expressed on leukocytes—particularly neutrophils and monocytes—where it regulates cell adhesion and migration [31, 32]. ScRNA-seq data from the TISCH2 NSCLC dataset (Supplementary Material 10, wjon.elmerpub.com) showed that *VNN2* expression was largely restricted to immune cell populations—especially monocytes and macrophages—while epithelial and malignant cells displayed minimal or nearly absent expression. Therefore, the reduced *VNN2* signal in bulk LUAD samples likely reflects decreased immune-cell infiltration or immunosuppressive remodeling of the TME, rather than tumor-intrinsic downregulation. Previous studies have mainly examined the role of *VNN2* in hematological disorders; for example, high *VNN2* expression has been associated with chemoresistance in pediatric B-cell acute lymphoblastic leukemia [33]. In solid tumors, limited evidence links *VNN2* to cancer progression—for instance, it has been included as a prognostic factor in hepatocellular carcinoma models, shown to regulate tumor invasion in osteosarcoma via miR-106a targeting, and associated with poor prognosis in metastatic renal cancer through high expression in peripheral myeloid cells [34–36]. Preliminary evidence also suggests that *VNN2* may influence tumor redox status and inflammatory pathways through its enzymatic activity, but mechanistic studies are scarce [37], particularly in the context of LUAD and the tumor immune microenvironment. Beyond tumor-intrinsic roles, recent studies have highlighted potential immunological functions of *VNN2*. Notably, its high expression in monocytic myeloid-derived suppressor cells (Mo-MDSCs) suggests a possible involvement in immunosuppressive states [38]. To date, however, no direct evidence has demonstrated that *VNN2* regulates NK-cell function. NK cells are central to immune surveillance in lung cancer, and their tumor infiltration correlates closely with patient prognosis [39]. While NK-cell dysfunction is a recognized mechanism of tumor immune evasion, the relationship between *VNN2*, NK cells, and LUAD risk has yet to be systematically examined. In this study, we integrated MR with sc-eQTL analysis to investigate whether genetically predicted *VNN2* expression was associated with LUAD risk across immune cell subtypes. This association was most pronounced in rNK cells, consistent with a potential cell type–specific role of *VNN2* in tumor immunity. By extending bulk-level omics analyses of lung cancer to single-cell resolution [40, 41], this work provides genetic evidence for a potential immunoregulatory role of *VNN2* that may inform future mechanistic studies on NK-cell–related immune pathways.

Together with previous evidence, our findings suggest that higher *VNN2* expression may be linked to a lower risk of LUAD, potentially by modulating the functional state of rNK cells and contributing to immune surveillance. *VNN2* encodes GPI-80, a GPI-anchored protein with pantetheinase activity, which has been implicated in the regulation of oxidative stress and inflammatory pathways, possibly through activation of the NF-κB and IL-1β pathways [42, 43]. Elevated *VNN2* expression has been associated with increased IL-1β levels and sustained NF-κB activation [44]. These pro-inflammatory mediators (e.g., IL-1β, type I interferons) could “prime” rNK cells [45], enhancing the expression of activating receptors (e.g., NKG2D, NKp44) and strengthening their capacity for tumor recognition. Resting NK cells serve as a reservoir for activated NK cells, and their abundance and activation capacity are critical for maintaining immune surveillance; however, excessive activation may indicate immune exhaustion. Epidemiological and experimental studies have linked higher NK-cell activity or tumor infiltration to reduced cancer risk and improved prognosis [46]. In addition, *VNN2* may influence NK-cell function indirectly by modulating immune-cell migration, adhesion, and metabolic state. For example, *VNN2* has been reported to regulate leukocyte β_2_-integrin (CD11b/CD18)–mediated adhesion and migration [47, 48], potentially facilitating NK-cell recruitment and retention within tumor tissues. Its pantetheinase activity generates cysteamine and pantothenic acid [45], which can modulate glutathione and reactive oxygen species (ROS) levels, possibly supporting NK-cell survival and activity under oxidative stress. While these mechanisms provide biologically plausible explanations for the observed cell type–specific genetic association, experimental validation—such as NK-cell co-culture assays and *in vivo* models—will be necessary to confirm their relevance.

This study has several limitations that should be acknowledged. First, although extensive sensitivity analyses were conducted, the MR results may still be susceptible to residual pleiotropy. Second, the genetic instruments were primarily derived from European-ancestry eQTL and pQTL datasets, which may limit the generalizability of our findings to other ancestries and lung tissue–specific regulatory landscapes. Third, compared with bulk eQTL analyses, single-cell eQTL–based MR was limited by smaller sample sizes, reduced statistical power, and potential biases in cell type annotation. Finally, although the association between *VNN2* and LUAD risk was most pronounced in rNK cells, the underlying biological mechanisms require experimental validation. Additionally, survival stratification according to NK cell–specific *VNN2* expression could not be evaluated because the available single-cell datasets lack patient follow-up information.

Furthermore, transcriptomic validation using the GEPIA2 database revealed no significant difference in overall survival between patients with high and low *VNN2* expression (log-rank P = 0.66; Supplementary Material 8, wjon.elmerpub.com), indicating that the protective genetic effect of *VNN2* on LUAD risk may not be directly reflected at the transcriptional level. In addition, single-cell transcriptomic analysis from the TISCH2 database showed that *VNN2* expression was predominantly enriched in immune cell populations—particularly monocytes and macrophages—whereas epithelial and malignant cells exhibited minimal expression (Supplementary Material 10, wjon.elmerpub.com). Although rNK cells were not individually annotated in this dataset, the immune cell–restricted expression pattern of *VNN2* is consistent with our sc-eQTL MR findings and supports its potential role in immune regulation within the LUAD microenvironment.

In addition, clinical factors such as tumor stage, grade, and treatment history were not adjusted in this study because the MR analyses were performed using publicly available summary-level data that lack individual-level clinical annotations. Nevertheless, the MR framework inherently mitigates potential confounding from these factors, as germline genetic variants are randomly allocated at conception and are independent of disease progression. Supporting evidence from the GEPIA2 stage analysis showed that *VNN2* expression levels did not significantly differ across LUAD stages (stage I–IV; ANOVA P = 0.964), suggesting that tumor stage heterogeneity is unlikely to bias our findings. Future studies integrating individual-level clinical data or implementing multivariable Mendelian randomization (MVMR) frameworks are warranted to further validate the causal relationship between *VNN2* expression and LUAD risk and to clarify its translational relevance in the clinical setting. This study focused on the genetic association between *VNN2* expression and LUAD susceptibility rather than its prognostic or predictive value. Future clinical and longitudinal studies are warranted to determine whether *VNN2* expression may serve as a biomarker for patient outcomes or therapeutic response.

### Conclusions

Multi-omics analyses indicated that genetically predicted higher *VNN2* expression was associated with a reduced risk of LUAD, most notably in rNK cells. These results highlight a potential cell type–specific role of *VNN2* in LUAD susceptibility and immune regulation. The findings should be regarded as hypothesis-generating, and further functional and clinical studies are needed to validate the biological mechanisms and assess any potential clinical relevance.

## Supplementary Material

Suppl 1 Summary of data sources and their analytical roles in the two-stage integrative framework.

Suppl 2 Two-sample MR analysis of blood eQTLs associated with LUAD (P < 0.05).

Suppl 3 Two-sample MR analysis of blood pQTLs associated with LUAD (P < 0.05).

Suppl 4 Differentially expressed genes between LUAD tumor and normal tissues (GEPIA2).

Suppl 5 Two-sample MR estimates of the association between genetically predicted *VNN2* expression and LUAD risk across 14 immune cell types.

Suppl 6 Summary of two-sample Mendelian randomization estimates for the association between genetically predicted *VNN2* expression/protein levels and LUAD risk using eQTL, pQTL, and sc-eQTL instruments.

Suppl 7 Leave-one-out sensitivity analyses for the association between *VNN2* and LUAD risk. (A) eQTL-based MR analysis. (B) pQTL-based MR analysis.

Suppl 8 Kaplan–Meier survival curve showing the association between *VNN2* expression and overall survival in LUAD patients based on TCGA data (GEPIA2).

Suppl 9 Expression of *VNN2* across pathological stages of lung adenocarcinoma (LUAD) based on TCGA data (GEPIA2 platform).

Suppl 10 Violin plot illustrating *VNN2* expression across major immune and stromal cell populations in NSCLC (GSE148071, TISCH2).

## Data Availability

All datasets used in this study are publicly available, including eQTLGen (whole-blood eQTL), deCODE (plasma pQTL), LUAD GWAS (European ancestry cohorts), GEPIA2 integrating TCGA and GTEx (transcriptomics), and OneK1K (single-cell eQTL). The analysis code and additional materials are available from the corresponding authors upon request.
