# Supplementary material for: Multi-Omics and Single-Cell Mendelian Randomization Reveal a Potential Role of VNN2 in Lung Adenocarcinoma in Resting Natural Killer Cells
Source: World J Oncol. 2026 Mar 5;17(2):247–55. doi: 10.14740/wjon2689 (PMC12978397; doi:10.14740/wjon2689)

**S7.** Leave-one-out sensitivity analyses for the association between *VNN2* and LUAD risk. (A) eQTL-based MR analysis. (B) pQTL-based MR analysis.


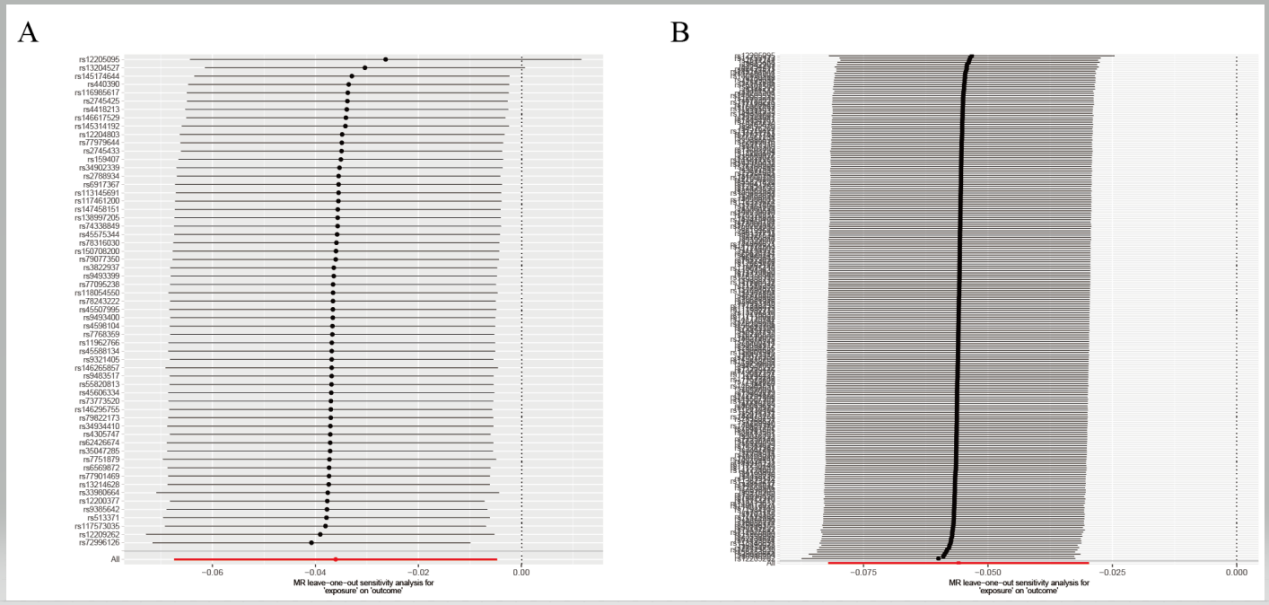

Supplement: Suppl 7 — Leave-one-out sensitivity analyses for the association between VNN2 and LUAD risk. (A) eQTL-based MR analysis. (B) pQTL-based MR analysis. [file wjon-17-02-247-s007.docx]
