# Supplementary material for: Multi-Omics and Single-Cell Mendelian Randomization Reveal a Potential Role of VNN2 in Lung Adenocarcinoma in Resting Natural Killer Cells
Source: World J Oncol. 2026 Mar 5;17(2):247–55. doi: 10.14740/wjon2689 (PMC12978397; doi:10.14740/wjon2689)
Supplement: Suppl 8 — Kaplan–Meier survival curve showing the association between VNN2 expression and overall survival in LUAD patients based on TCGA data (GEPIA2). [file wjon-17-02-247-s008.docx]

**S8. Kaplan–Meier survival curve showing the association between *VNN2* expression and overall survival in LUAD patients based on TCGA data (GEPIA2).**

**
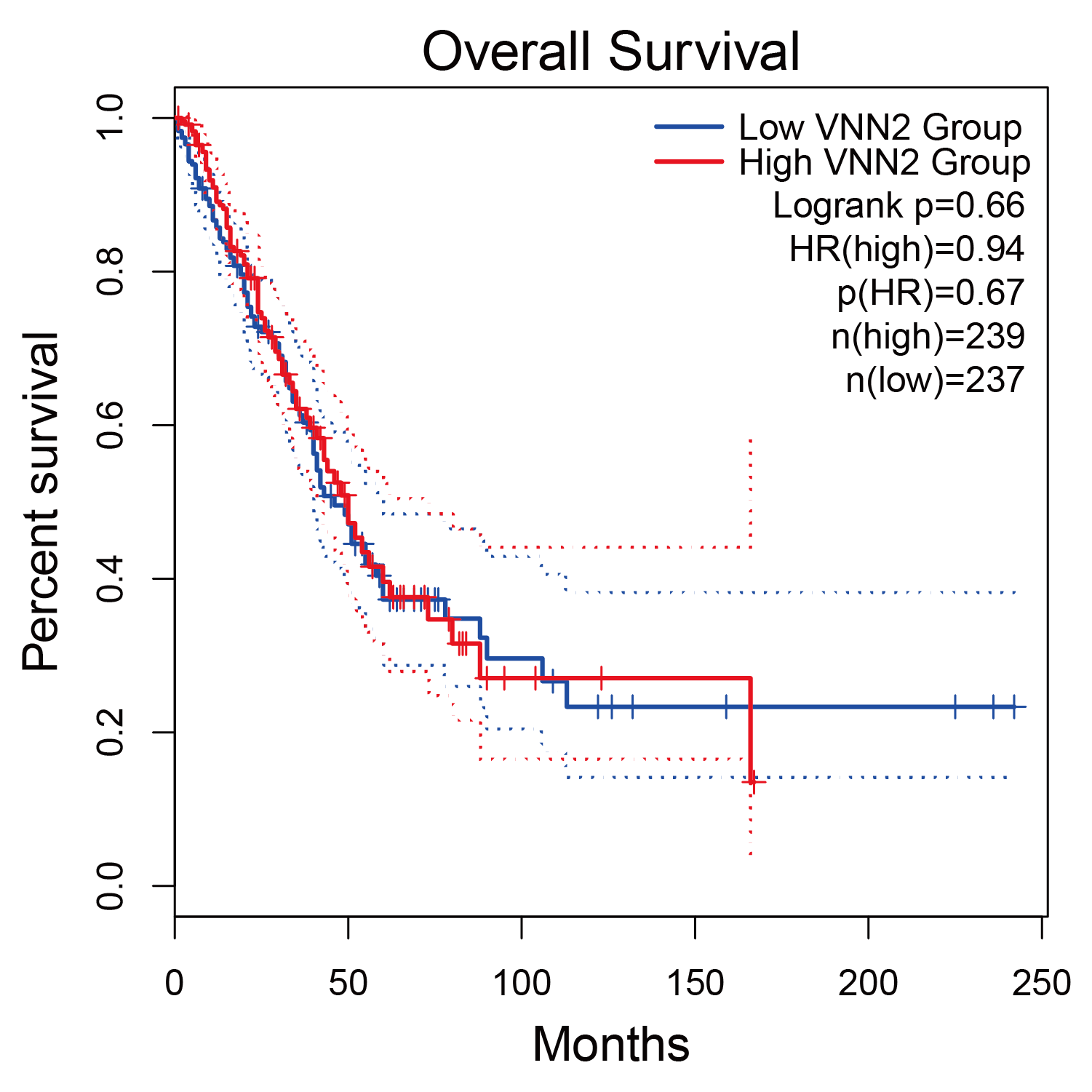
**
