# Supplementary material for: Multi-Omics and Single-Cell Mendelian Randomization Reveal a Potential Role of VNN2 in Lung Adenocarcinoma in Resting Natural Killer Cells
Source: World J Oncol. 2026 Mar 5;17(2):247–55. doi: 10.14740/wjon2689 (PMC12978397; doi:10.14740/wjon2689)
Supplement: Suppl 9 — Expression of VNN2 across pathological stages of lung adenocarcinoma (LUAD) based on TCGA data (GEPIA2 platform). [file wjon-17-02-247-s009.docx]

**S9. Expression of *VNN2* across pathological stages of lung adenocarcinoma (LUAD) based on TCGA data (GEPIA2 platform).**

**
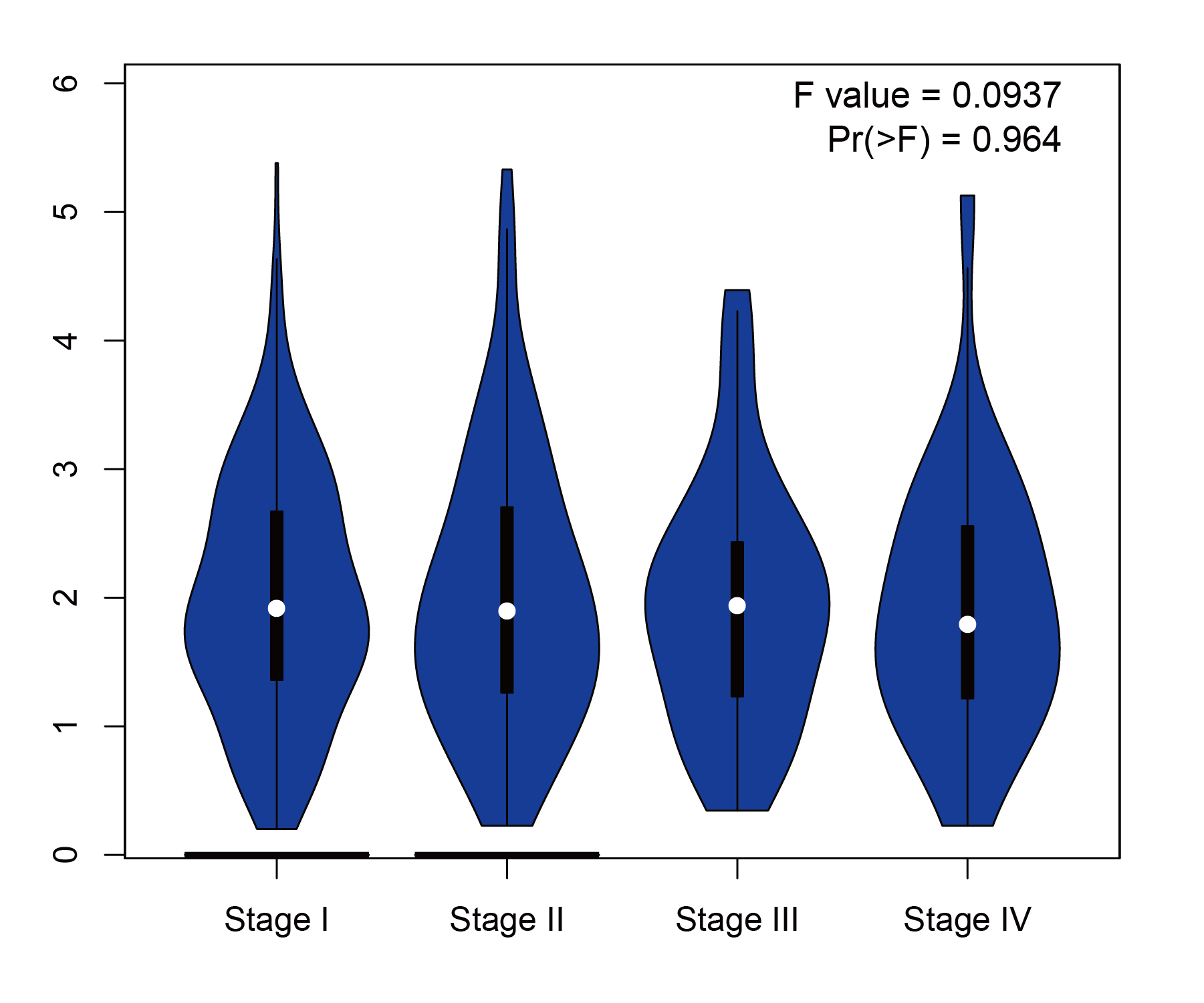
**
