# Supplementary material for: Multi-Omics and Single-Cell Mendelian Randomization Reveal a Potential Role of VNN2 in Lung Adenocarcinoma in Resting Natural Killer Cells
Source: World J Oncol. 2026 Mar 5;17(2):247–55. doi: 10.14740/wjon2689 (PMC12978397; doi:10.14740/wjon2689)
Supplement: Suppl 10 — Violin plot illustrating VNN2 expression across major immune and stromal cell populations in NSCLC (GSE148071, TISCH2). [file wjon-17-02-247-s010.docx]

**S10. Violin plot illustrating *VNN2* expression across major immune and stromal cell populations in NSCLC (GSE148071, TISCH2).**

**
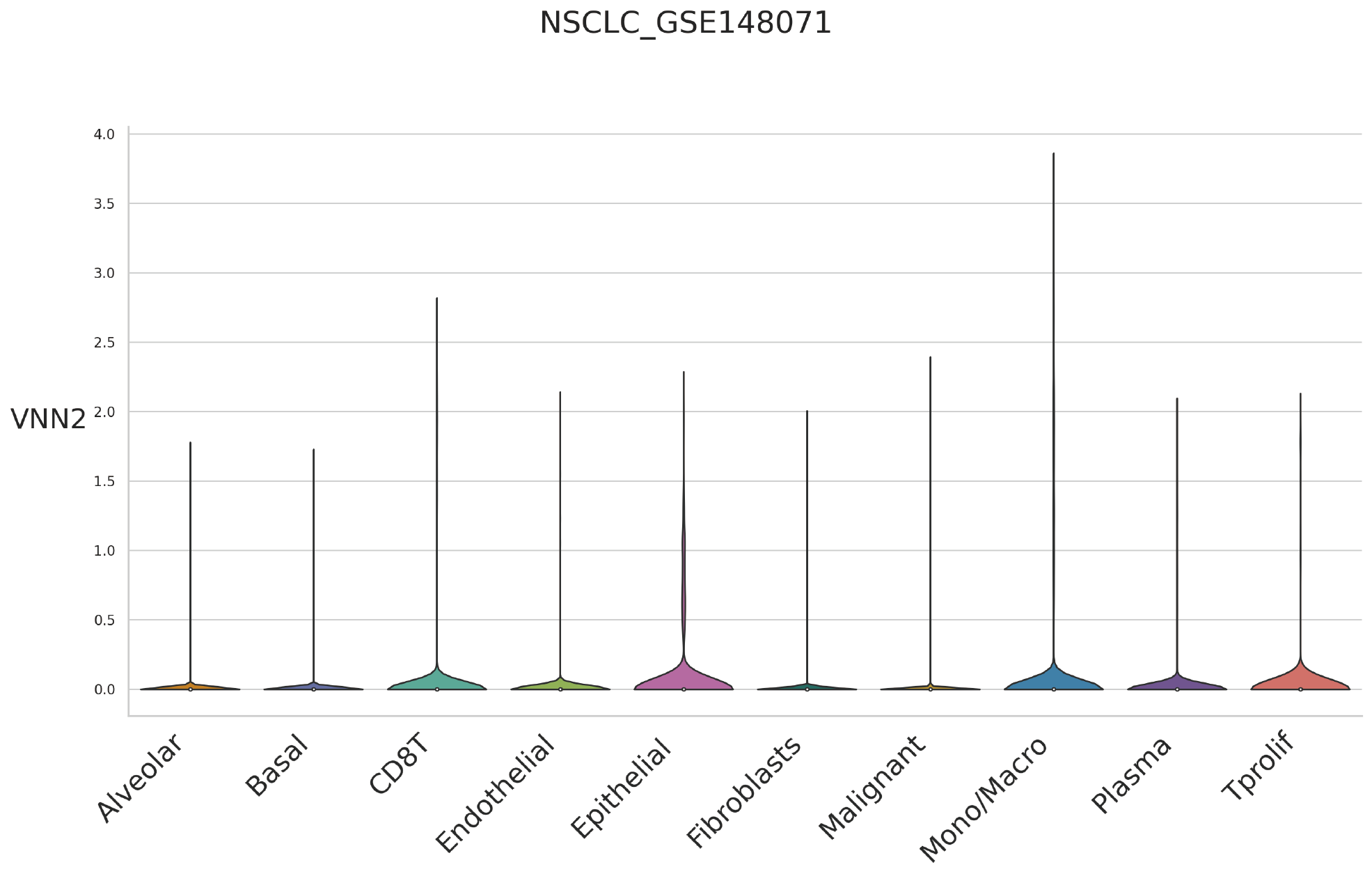
**
